# Effect of low-frequency dorsal root ganglion stimulation in the treatment of chronic pain

**DOI:** 10.1007/s00701-023-05500-1

**Published:** 2023-01-27

**Authors:** G. S. Piedade, S. Gillner, P. S. McPhillips, J. Vesper, P. J. Slotty

**Affiliations:** 1 Department of Neurosurgery, Helios Universitätsklinikum Wuppertal, Universität Witten/Herdecke, Heusnerstr. 40, 42283 Wuppertal, Germany; 2 Department of Neurosurgery, Heinrich-Heine-Universität Düsseldorf, Düsseldorf, Germany; 3 Department of Functional Neurosurgery and Stereotaxy, Heinrich-Heine-Universität Düsseldorf, Düsseldorf, Germany

**Keywords:** Neuropathic pain, Dorsal root ganglion stimulation, Frequency, Neuromodulation, Nociceptive pain

## Abstract

**Background:**

The role of stimulation parameters, especially stimulation frequency is not well understood in dorsal root ganglion stimulation. Previous studies documented higher effectiveness for frequencies as low as 20 Hz, but there is evidence that even lower values could lead to better outcomes. In this study, we investigate the influence of low-frequency DRG-S.

**Method:**

This is a randomized double-blind clinical trial with a crossover design. Patients with an already implanted DRG-S system were included and randomly tested with 4 Hz, 20 Hz, 60 Hz, and sham stimulation. Amplitude was adjusted to subthreshold values for each frequency. Each frequency was tested for 5 days, followed by a 2-day washout period. Patients were assessed using VAS, McGill Pain Questionnaire, EQ-5D-5L, and Beck Depression Inventory.

**Results:**

Seventeen patients were in included. Time between inclusion in this study and primary implant was 32.8 months. Baseline stimulation frequency was 20 Hz in all patients. Mean baseline pain intensity was VAS 3.2 (SD 2.2). With 4-Hz stimulation, VAS was 3.8 (SD 1.9), with 20 Hz VAS 4.2 (SD 2.0) and with 60 Hz VAS 4.6 (SD 2.7). Worst pain control was seen with sham stimulation with a VAS of 5.3 (SD 3.0). Stimulation with 4 Hz achieved lower VAS scores, but this was only statistically significant when compared to sham (*p* = 0.001). A similar trend favoring 4-Hz stimulation was seen using the Beck Depression Inventory, but in this case no statistical significance was found. Outcomes of McGill Pain Questionnaire and EQ-5D-5L favored 20 Hz stimulation, but again without statistical significance.

**Conclusions:**

Low-frequency stimulation was not significantly better than classic 20-Hz stimulation in relieving pain intensity; the study might however be underpowered. Longer washout and observational periods might also be necessary to show clear differences in frequency response.

## Introduction

Over the past years, dorsal root ganglion stimulation (DRG-S) has become a key instrument in neuromodulation for chronic neuropathic pain. The role of stimulation parameters, especially of stimulation frequency, is less well understood compared to classic spinal cord stimulation (SCS), likely due to the new target structure and the relative newness of the method. We published a first randomized double-blind clinical trial assessing the effect of different stimulation frequencies in DRG-S and demonstrated the superiority of stimulation with 20 Hz over frequencies of 40 Hz, 60 Hz, and 80 Hz [5]. A previous animal study using 1 Hz [4], however, raised the hypothesis that even lower stimulation frequencies could possibly lead to better outcomes. Dr. Chapman and his team were the first to document the effect of 4 Hz in humans in a landmark case series [2], but data from a randomized controlled study are missing. We therefore continued the clinical trial with an altered design to deliver high-quality data on the effect of low-frequency stimulation in the treatment of chronic pain.

## Material and methods

Adult patients using DRG-S and followed up at the Department of Functional Neurosurgery and Stereotaxy of the Heinrich-Heine-University Düsseldorf were invited to participate in the study. Significant pain possibly confounding the study results was an exclusion criterion. Informed consent was obtained. The study was originally approved by the Ethics Committee of the Medical Faculty under the number 2020–1120; the new study design including low-frequency stimulation was approved with an amendment. Registration in the German Clinical Trials Register (DRKS) is under DRKS00022557.

Neuropathic pain was assessed with PainDetect (0–38 points) at the baseline, when all patients were being stimulated with 20 Hz. All subjects tested in a randomized order four different settings of stimulation parameters: stimulation frequencies of 4 Hz, 20 Hz, 60 Hz, and sham stimulation. Stimulation amplitude was individually optimized in each case, so that stimulation was at subthreshold level for the entire duration of the study. Subjects and investigators were blinded; a study nurse had access to unblinded data. Each stimulation setting was tested for 5 days and followed by a 2-day washout period. There was also a washout period before the study starts. When patients reported intolerable pain without stimulation, the washout period was shortened to a single day. In case of intolerable pain during a test period, the testing was shortened. A study nurse programmed stimulation parameters in advance, so that patients could randomly change them each week at home. At the end of each phase, patients were interviewed by phone and completed numbered questionnaires.

During the study, evaluation of pain intensity and quality was done using the visual analog scale and McGill Pain Questionnaire (MPQ, 0–78 points); the prevalence of depression was assessed with the Beck Depression Inventory (BDI, 0–63 points) and the quality of life with EQ-5D-5L (index 0–1).

Repeated measurement one-way ANOVA was used for comparison between baseline data and measurements at the different frequency settings; Tukey’s test was selected for post hoc analysis.

## Results

Twenty-three patients were pre-selected and asked to participate in the study; eighteen agreed but one patient was excluded because of another pain syndrome acting as a confounding factor. Seventeen patients were included in the study; the group had a mean of 55.2 years old (range: 29–76) and was under DRG-S for an average of 32.8 months (range: 2–120) (Table 1). Eight subjects scored over 12 in PainDetect at the baseline, meaning a high probability of neuropathic pain. Most common indications for DRG-S were CRPS (6 subjects) and postsurgical pain (5), followed by intercostal neuralgia (2). All subjects were under stimulation with 20 Hz as a standard; they were satisfied with the therapy and had an adequate coverage of the painful area. The mean VAS at the baseline was 3.2 (SD 2.2), McGill Pain Questionnaire resulted in 7.7 points (SD 5.4), EQ-5D-5L score was 0.82 (SD 0.10), and Beck Depression Inventory resulted in 6.9 points (SD 5.9). Data were complete for all patients except in two cases, because the subjects could not undergo the phase of sham stimulation due to unacceptable pain.

**Table 1:** Pain intensity in the baseline and under stimulation frequencies of 4 Hz, 20 Hz, 60 Hz, and sham stimulation

No	Age	Pain etiology	Time under DRG-S (mo)	Pain detect	VAS baseline	VAS 4 Hz	VAS 20 Hz	VAS 60 Hz	VAS Sham
1	58	Postmastectomy	14	14	2	7	7	3	7
2*	49	Traumatic nerve injury	94	29	6	6	7	9**	9**
3	51	Intercostal neuralgia	6	0	1	1	1	1	1
4	62	Postsurgical after implantation of joint prothesis	13	20	5	4	4	4	4
5*	37	CRPS I	18	18	6	6	5	10**	-**
6	72	Intercostal neuralgia	13	2	3	4	4	5	3
7*	76	Diabetic polyneuropathy	18	13	8	5	7	8	10**
8	75	Polyneuropathy after chemotherapy	83	17	4	3	6	3	4
9*	61	Postsurgical after implantation of joint prothesis	120	12	2	4	5	6	9
10	54	Postherpetic neuralgia	10	12	2	3	5	2	3
11	72	CRPS II	39	6	3	4	3	6	4
12	59	CRPS I	4	7	3	5	5	5	5
13	58	Postsurgical after ankle fracture	26	8	1	3	4	6	8
14*	36	CRPS II	31	16	3	5	4	5	8
15	29	CRPS II	2	3	0	1	0	1	2
16	35	Postsurgical after tarsal tunnel release	64	10	0	0	2	2	3
17	55	CRPS II	4	16	5	4	3	3	-**
		Mean (SD)	32.9 (35.7)	11.9 (7.3)	3.2 (2.2)	3.8 (1.9)	4.2 (2.0)	4.6 (2.7)	5.3 (3.0)

^*^Patients who shortened washout phases due to intolerable pain. **Test phase was shortened by the patient because of intolerable pain. *CRPS*, complex regional pain syndrome; *SD*, standard deviation

**Fig. 1:**
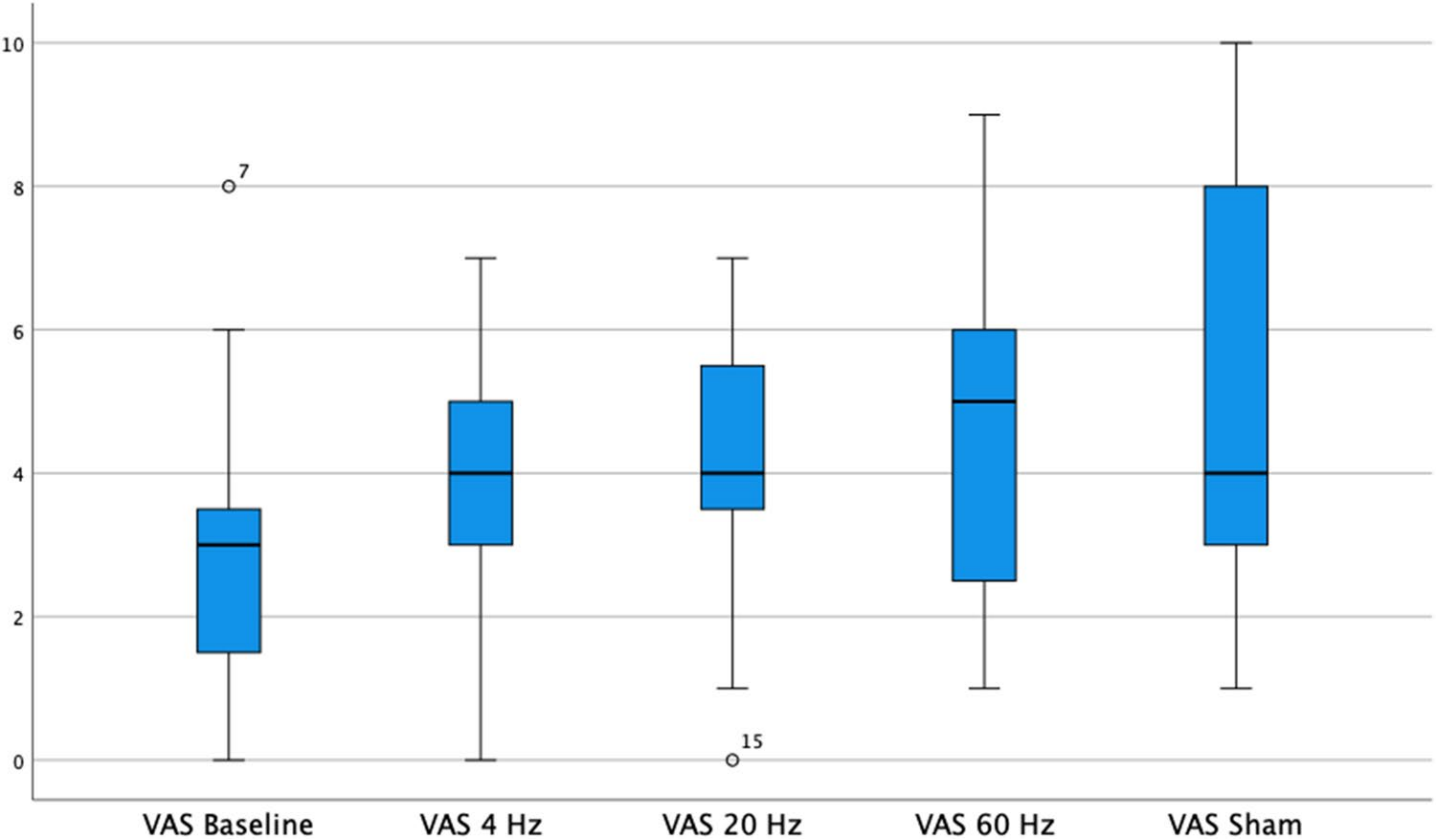
Pain intensity in the visual analog scale under different stimulation frequencies

Pain intensity scores in the VAS achieved for 4 Hz, 20 Hz, 60 Hz, and sham stimulation were 3.8 (SD 1.9), 4.2 (SD 2.0), 4.6 (SD 2.7), and 5.3 (SD 3.0) respectively (Fig. 1). The baseline scores were not significantly different from those with 4-Hz stimulation (*p* = 0.492), but from scores under stimulation with 20 Hz (*p* = 0.048), 60 Hz (*p* = 0.024), and sham (*p* < 0.001). Although achieving lower pain intensity scores, stimulation with 4 Hz was not significantly different from 20 Hz (*p* = 0.743) and 60 Hz (*p* = 0.577), only from sham (*p* = 0.017). Stimulation with 20 Hz did not differ significantly from 60 Hz nor sham.

**Fig. 2:**
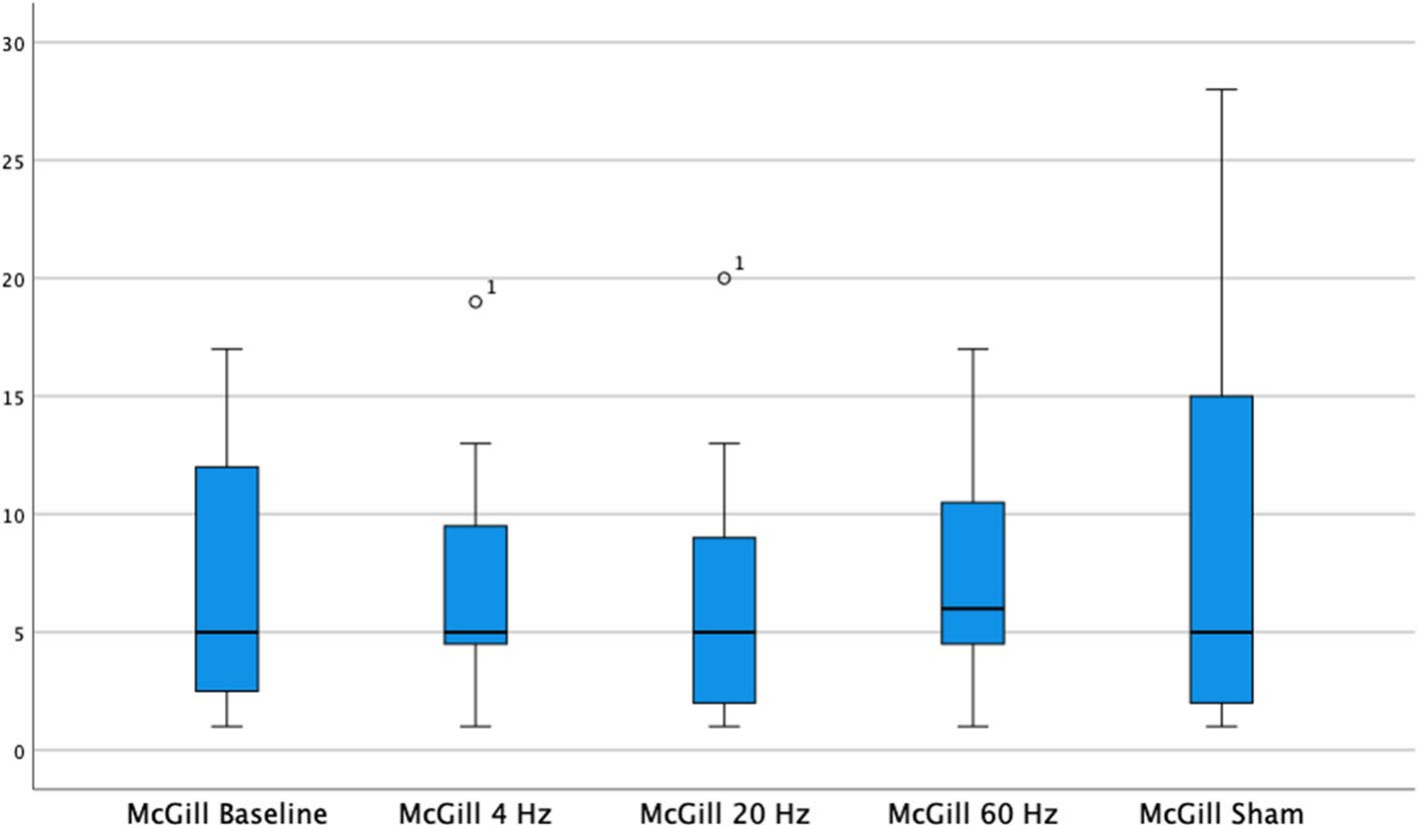
Results of McGill Pain Questionnaire under different stimulation frequencies

**Fig. 3:**
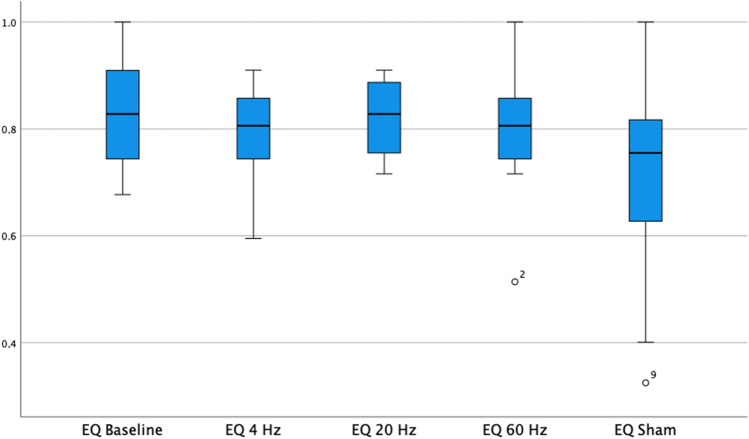
Results of EQ-5D-5L under different stimulation frequencies

McGill Pain Questionnaire resulted for the same groups 8.0 (SD 5.4), 6.7 (SD 5.4), 8.9 (SD 7.5), and 8.6 (SD 8.4) points (Fig. 2). For this parameter, no statistical significance was found considering all test phases in Tukey’s test. The assessment of quality of life with EQ-5D-5L indexes resulted in 0.75 (SD 0.23), 0.79 (SD 0.15), 0.74 (SD 0.26), and 0.72 (SD 0.19) (Fig. 3). No statistical significance was achieved among the groups. Both in McGill Pain Questionnaire and in EQ-5D-5L, stimulation with 20 Hz, achieved better results, but not statistically significant.

**Fig. 4:**
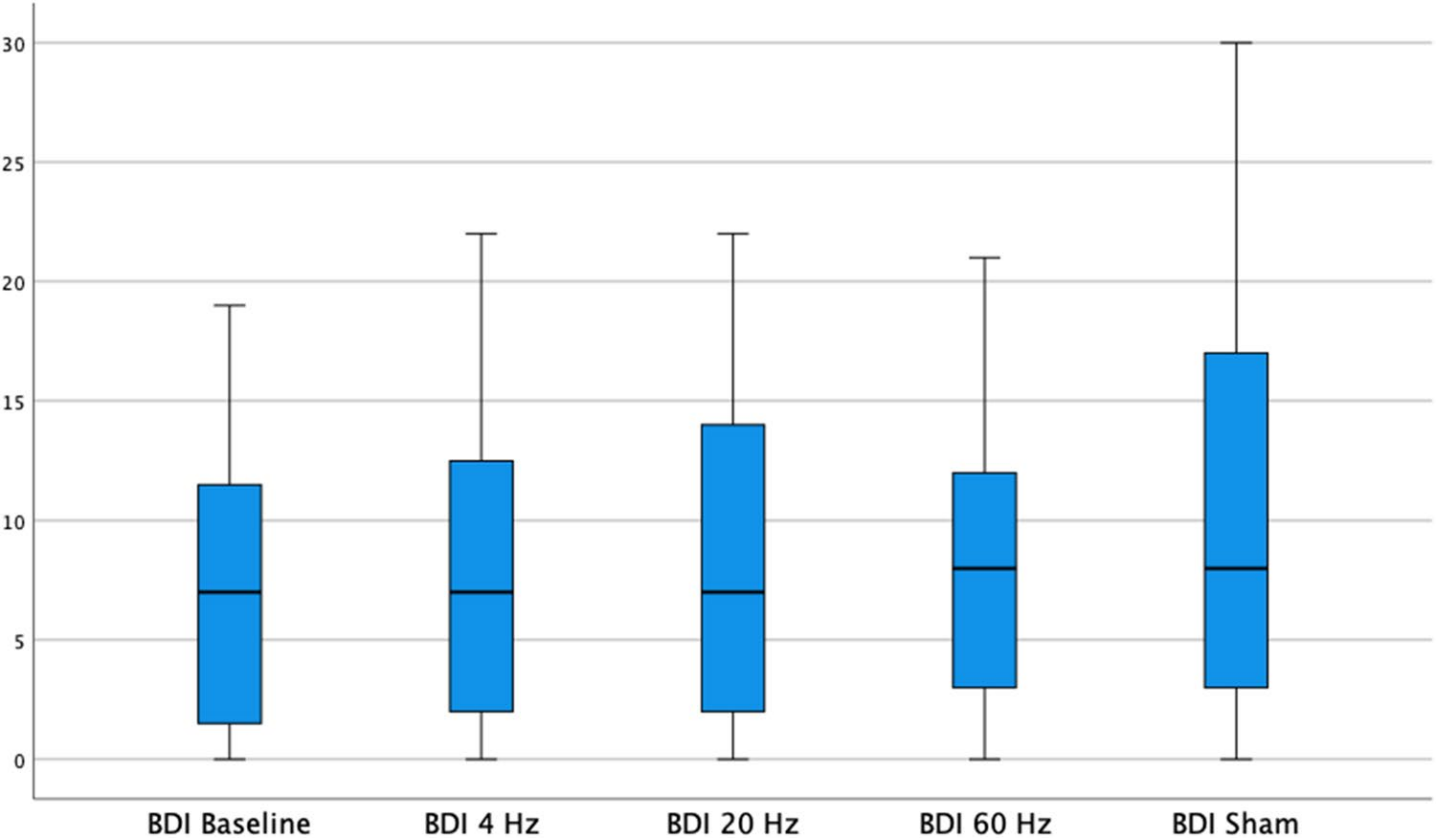
Results of the Beck Depression Inventory under different stimulation frequencies

Beck Depression Inventory resulted in 9 (SD 7.3), 9.1 (SD 7.7), 9.4 (SD 7.7), and 10.5 (SD 9.1) points (Fig. 4). Repeated measurement one-way ANOVA did not indicate statistical significance in this case as well.

## Discussion

Although stimulation with 4 Hz showed an interesting trend eliciting a better pain relief than any other tested frequencies in a blinded fashion, this difference was not statistically significant in this study. The same trend was seen using the Beck Depression Inventory, but results from McGill Pain Questionnaire and EQ-5D-5L favored stimulation with 20 Hz—again without statistical significance. This second phase of the original clinical trial was underpowered due to the sample size and possibly also because the effect of 4-Hz stimulation might be only discretely better than that of 20-Hz stimulation. Similar to the important case series of Dr. Chapman and his team tapering stimulation frequencies from 16 to 4 Hz, no statistical significance was found when comparing pre- and post-tapering pain intensity scores in a group of 20 patients [2]. There is, however, evidence from experimental studies in animals favoring stimulation with lower stimulation frequencies—not necessarily because of a better effect over pain intensity scores.

Dorsal root ganglion stimulation possibly acts activating low-threshold mechanoreceptors, which report fine touch sensation to the spinal cord and potentially reduce the perception of pain [3]. The fact that stimulation frequencies over 20 Hz achieve worse pain intensity scores [5] might be explained by phase locking—the neural tissue’s property to fire simultaneously with the stimulation frequency. This occurs up to a frequency limit depending on electrophysiological properties of the target fibers, but for the case of low-threshold mechanoreceptors of rats this corresponds to a frequency limit of 20 Hz [1].

The reason why even lower stimulation frequencies might be more effective than 20 Hz itself could be the long-term depression of synaptic transmission in the substancia gelatinosa, a phenomenon once described in rats by Sandkühler et al. His team stimulated dorsal roots with 1-Hz stimulation for 15 min and measured the amplitude of excitatory postsynaptic potentials in the substancia gelatinosa. When the intensity of conditioning stimulation was raised to 10 V, recruiting a maximum of Aδ fibers, there was a robust long-term depression of synaptic transmission for the entire duration of the recording, that lasted for 160 min [6]. This interesting finding may also explain the results obtained by Dr. Koetsier et al., who tested stimulation frequencies of 1 Hz, 20 Hz, and 1000 Hz in the DRG of rats with diabetic polyneuropathy. Once again, the lowest stimulation frequency did not achieve statistical significance regarding the relief of mechanical hypersensitivity. However, the effect of stimulation with 1 Hz lasted for more than 60 min after cessation of stimulation, while the return to the baseline occurred much earlier under 20 Hz and 1000 Hz [4].

Low-frequency stimulation of the DRG might not only have a long-lasting effect, but also intuitively reduces the amount of electrical charge used by the patients and could possibly mean a relevant extension of battery lifetime. Further clinical trials on the issue should consider these characteristics when assessing the effect of low-frequency stimulation and maybe use longer test phases and longer washout periods. Larger trials could even be able to identify a significant difference in pain intensity scores, possibly confirming the clear trend of this study favoring lower stimulation frequencies. Ideally, a study with a large sample size could also analyze the effect of different frequencies in each pain etiology, as different entities target different nerve fibers and are therefore possibly better addressed by specific frequency ranges.

Although the results were not statistically significant, the test of different stimulation frequencies should be offered to individual patients under DRG-S seeking better results. Changes in pain relief are sometimes dramatic in sensitive patients, but a thorough evaluation could only be done in the course of weeks and assessing not only pain intensity scores, but also indicators of quality of life. Even when lower stimulation frequencies provide similar results to standard 20-Hz stimulation in the individual patient, we would recommend the lowest efficient frequency with a view to saving battery lifetime.

## Conclusions

Low-frequency stimulation was not significantly better than classic 20-Hz stimulation in relieving pain intensity; the study might however be underpowered. Longer washout and observational periods might be necessary to show clear differences in frequency response.

## Abbreviations

BDI: Beck Depression Inventory
CRPS: Complex regional pain syndrome
DRG-S: Dorsal root ganglion stimulation
MPQ: McGill Pain Questionnaire
SCS: Spinal cord stimulation
VAS: Visual analog scale
